# Infrared and Visual Image Fusion through Fuzzy Measure and Alternating Operators

**DOI:** 10.3390/s150717149

**Published:** 2015-07-15

**Authors:** Xiangzhi Bai

**Affiliations:** 1 Image Processing Center, Beijing University of Aeronautics and Astronautics, Beijing 100191, China; E-Mail: jackybxz@buaa.edu.cn; Tel.: +86-10-8233-8578; 2 State Key Laboratory of Virtual Reality Technology and Systems, Beihang University, Beijing 100191, China

**Keywords:** alternating operator, toggle operator, mathematical morphology, fuzzy measure

## Abstract

The crucial problem of infrared and visual image fusion is how to effectively extract the image features, including the image regions and details and combine these features into the final fusion result to produce a clear fused image. To obtain an effective fusion result with clear image details, an algorithm for infrared and visual image fusion through the fuzzy measure and alternating operators is proposed in this paper. Firstly, the alternating operators constructed using the opening and closing based toggle operator are analyzed. Secondly, two types of the constructed alternating operators are used to extract the multi-scale features of the original infrared and visual images for fusion. Thirdly, the extracted multi-scale features are combined through the fuzzy measure-based weight strategy to form the final fusion features. Finally, the final fusion features are incorporated with the original infrared and visual images using the contrast enlargement strategy. All the experimental results indicate that the proposed algorithm is effective for infrared and visual image fusion.

## 1. Introduction

Different imaging sensors produce images with different degrees of superiority [1,2,3,4,5,6,7,8,9]. Infrared imaging sensors produce images with important regions which could not be observed by visual imaging sensors. Visual images contain rich details which could not be provided by the infrared image. An effective and useful way to produce an image with important regions and rich details is to fuse the infrared and visual image.

The image regions in infrared images and the rich details in visual images are the spatial information. Infrared and visual image fusion should effectively combine these spatial features to produce a clear fusion result with rich details. The crucial issue of infrared and visual image fusion is how to effectively extract the image features, including the image regions and details. Combining these features into the final fusion result would produce a clear fusion image. To achieve this purpose, many algorithms have been proposed [10,11,12,13,14,15,16,17,18,19,20,21,22,23,24]. A direct averaging algorithm is simple and easy to implement [10,11], but image details may be heavily smoothed, which cannot produce a clear fusion image with rich details. Wavelet, curvelet and shearlet transforms [12,13,14,15,16] extract image features through the pyramid decomposition of the original infrared and visual images. However, some useful image information may be lost, which may produce unclear fusion results. Segmentation-based algorithms [17,18,19] are also used for image fusion, but, the effective segmentation results which may affect the fusion result cannot be obtained easily. Independent component analysis, principal component analysis or Laplacian pyramid-based algorithms [20,21,22] extract the main information of the original infrared and visual images to produce the fusion image, but again, some image information is lost, which may result in an unclear fusion image. Neural networks and some intelligent tools [23,24] were also tried for image fusion. However, most of them are mainly used for multi-focus image fusion.

Mathematical morphology has been the important theory in the field of image analysis [25,26,27,28,29,30,31,32], which is also used for infrared and visual image fusion [4,5,10,25]. Using the pyramid decomposition strategy based on the morphological operators is useful for image fusion [26,27]. Although a clear fusion image may be produced, some image details may be still smoothed and some artifacts may also be produced. This would affect the further analysis of the fusion result. Top-hat transforms have been used or improved for the fusion of infrared and visual images [5,10,29,32]. However, some image details of the original images may be not well preserved in the final fusion image. Toggle operators using opening and closing as primitives was also used for infrared and visual image fusion [4], which could preserve image details in the final fusion image, but some image details are still smoothed. In all, most of the existing algorithms may not perform well for producing a clear fusion result with rich details.

Morphological alternating filters [26,27], which are the classical alternating operators, are defined as alternatively operating the morphological opening and closing operators [26,27,30,31]. Then, both the bright and dark image features could be identified by the alternating filters. However, because of the defect of smoothing useful image information, the classical alternating filters may not effectively identify some useful image features or may produce noise in the resulting image. This would affect the performance of infrared and visual image fusion. Since the alternating operators are effective morphological operators, a new way of constructing the novel alternating operators with more effective performance for feature extraction has been proposed [33]. The constructed alternating operators using the opening and closing based toggle operator could effectively extract the spatial features, including the image regions and details. These features could be used for fusion, which may produce a clear fusion result with rich details. Moreover, combining the multi-scale features in the morphological operator-based algorithm is one important step. The fuzzy measure, linear index of fuzziness [34,35,36,37,38,39] used in this paper, is defined based on the spatial information of images, which could be used to quantify the importance of the multi-scale spatial features. Then, using the fuzzy measure, the important multi-scale spatial features could be effectively combined.

Based on the analysis above, an effective algorithm for infrared and visual image fusion by using the fuzzy measure and constructed alternating operators is demonstrated in this paper. Firstly, based on the analysis of the constructed alternating operators using opening and closing based toggle operators, two types of alternating operators are used for extracting the multi-scale fusion features. Secondly, the extracted multi-scale fusion features are combined through the fuzzy measure based weight strategy to form the final fusion features. Finally, the final fusion image is produced by adjusting the contrast of the final fusion features. All the experimental results indicate that because the alternating operators could effectively extract the features for fusion and the fuzzy measure could effectively fuse the features, the proposed algorithm performs effectively for infrared and visual image fusion.

## 2. Mathematical Morphology

### 2.1. Basic Morphological Operators

Many of the morphological operators are the useful tool for different applications [25,26,27,28,29,30,31], which are usually defined based on two sets: the original image *f* (*x, y*) and structuring element *B* (*u, v*). The pixel coordinates of *f* and *B* are represented by (*x, y*) and (*u, v*), respectively. Two of the basic morphological operators, dilation (⊕) and erosion (Θ), are defined using *f* and *B* as follows:

(1) $$f\;⊕B\;=\;\underset{u,v}{\max}(f(x-u,y-v)+B(u,v))$$

(2) $$f\;\Theta B\;=\;\underset{u,v}{\min}(f(x+u,y+v)-B(u,v))$$

Two important morphological operators, opening and closing (denoted by f∘B and f•B), are defined by composing the morphological dilation and erosion as follows:

f∘B = (f ΘB) ⊕B

f•B = (f ⊕B) ΘB

### 2.2. Toggle Operator

Toggle operators are defined based on the results of morphological operators following different pre-defined rules. One toggle operator defined based on the opening and closing operator is as follows [32]:

TO (f)(x, y) = 

$$\{\begin{array}{c} f\circ B(x,y),\text{ if }f•B(x,y)-f(x,y)<f(x,y)-f\circ B(x,y)\\ f•B(x,y),\text{ if }f•B(x,y)-f(x,y)>f(x,y)-f\circ B(x,y)\\ f(x,y),\;else \end{array}$$

Opening and closing smooth the bright and dark image features, which would change the gray values of these features. This definition of toggle operator indicates that the smoothed image features by opening or closing with larger gray value changes would be retained in the toggle operator result. These remaining image features usually represent the important features in the images [4,32].

## 3. Alternating Operator by Opening and Closing Based Toggle Operator

### 3.1. Basic Operator

Because of the smoothing by opening, the identified bright image features by *TO* would have smaller gray values than the corresponding pixels of the original image. Thus, the identified bright image features by *TO* could be obtained as follows [4,32]:

$$IFB_{B}\left(f\right)\left(x,\;y\right)=\max(f(x,y)-TO_{B}(f)(x,y),0)$$

*IFB* contains the bright image features, which has similar properties as the morphological opening operator [32]. Similarly, the identified dark image features by *TO* could be obtained as follows [4,32]:

$$IFD_{B}\;\left(f\right)\left(x,\;y\right)\;=\;\max(TO_{B}(f)(x,y)-f(x,y),0)$$

*IFD* contains the dark image features, which has similar properties as the morphological closing operator [32].

### 3.2. Multi-Scale Extension

Multi-scale structuring elements could be used by morphological operators to extract the multi-scale image features. Suppose *B*_1_, …, *B_n_* be a sequence of multi-scale structuring elements. *B_i_* represents the structuring element at scale *i*, 1 ≤ *I* ≤ *n.* Through utilizing the structuring element *B_i_* at scale *i*, the multi-scale expression of toggle operator is as follows [4,32]:

$$TO_{B_{i}}(x,\;y)\;=\;$$

$$\{\begin{array}{c} f\circ B_{i}(x,y),\text{ if }f•B_{i}(x,y)-f(x,y)<f(x,y)-f\circ B_{i}(x,y)\\ f•B_{i}(x,y),\text{ if }f•B_{i}(x,y)-f(x,y)>f(x,y)-f\circ B_{i}(x,y)\\ f(x,y),\;else \end{array}$$

By using the multi-scale toggle operator $TO_{B_{i}}$, the multi-scale expressions of *IFB* and *IFD* are as follows [4,32]:

$$IFB_{B_{i}}\left(f\right)\left(x,\;y\right)\;=\;\max(f(x,y)-TO_{B_{i}}(f)(x,y),0)$$

$$IFD_{B_{i}}\;\left(f\right)\left(x,\;y\right)\;=\;\max(TO_{B_{i}}(f)(x,y)-f(x,y),0)$$

### 3.3. Alternating Operators

*IFB* and *IFD* have similar properties as the morphological opening and closing operators, respectively. Utilizing a strategy similar to constructing the alternating filters through alternatively operating the opening and closing, the alternating operators through alternatively operating the *IFB* and *IFD* could be defined as follows [33]:

$$AO_{i}^{1}(x,y)\;=\;IFD_{B_{i}}(IFB_{B_{i}}\left(x,y)\right)$$

$$AO_{i}^{2}(x,y)\;=\;IFB_{B_{i}}(IFD_{B_{i}}\left(x,y)\right)$$

$$AO_{i}^{3}(x,y)\;=\;IFB_{B_{i}}(IFD_{B_{i}}(IFB_{B_{i}}\left(x,y))\right)$$

$$AO_{i}^{4}(x,y)\;=\;IFD_{B_{i}}(IFB_{B_{i}}(IFD_{B_{i}}\left(x,y))\right)$$

Because *IFB* and *IFD* smooth the bright and dark image features, the constructed alternating operators sequentially smooth the bright and dark image features at different scales, which indicates that the constructed alternating operators could be used to identify the image features at different scales. This would be useful for different image analysis applications.

## 4. Infrared and Visual Image Fusion

### 4.1. Multi-Scale Fusion Feature Extraction

The two types of alternating operators $AO_{i}^{1}$ and $AO_{i}^{2}$ alternatively operate the morphological opening and closing operators, which could both smooth the important bright and dark image features. In infrared and visual images, the effective featrues are bright or dark features. This means that these two types of alternating operators $AO_{i}^{1}$ and $AO_{i}^{2}$ could be used to extract both the bright and dark features, which would be helpful for the infrared and visual image fusion.

Because $AO_{i}^{1}$ and $AO_{i}^{2}$ could both smooth the bright and dark image features, the gray values of these image features are different compared to the gray values of these image features in the original image. Thus, extracting image features through comparing the gray values of the result of morphological operators and the original infrared or visual images [33] would be also effective for extracting the features for infrared and visual image fusion.

Let *f* and *g* represent the original infrared and visual images for fusion. For infrared image *f*, bright image features having large gray values may become small after the smoothing by alternating operator $AO_{i}^{1}$ following the increasing of the scale numbers. Thus, by using the first type of alternating operator $AO_{i}^{1}$, the identified bright features of the original infrared image corresponding to scale *i* could be expressed as follows:

$$BFAO_{i}^{1}(f)\;=\;\max[f(x,y)-[{\text{AO}}_{i}^{1}(f)](x,y),0]$$

Also, by using the second type of alternating operator $AO_{i}^{2}$, the identified bright features of the original infrared image corresponding to scale *i* could be expressed as follows:

$$BFAO_{i}^{2}(f)\;=\;\max[f(x,y)-[{\text{AO}}_{i}^{2}(f)](x,y),0]$$

The bright features of the original infrared image *f* extracted by the two types of the alternating operators could be calculated as the combination of $BFAO_{i}^{1}(f)$ and $BFAO_{i}^{2}(f)$ as follows:

$$BFAO_{i}(f)\;=\;[BFAO_{i}^{1}(f)\;+\;BFAO_{i}^{2}(f)]/2$$

In the same way, the bright features of the original visual image *g* extracted by the two types of the alternating operators could be calculated as follows:

$$BFAO_{i}(g)\;=\;[BFAO_{i}^{1}(g)\;+\;BFAO_{i}^{2}(g)]/2$$

where:

$$BFAO_{i}^{1}(g)\;=\;\max[g(x,y)-[{\text{AO}}_{i}^{1}(g)](x,y),0]$$

$$BFAO_{i}^{2}(g)\;=\;\max[g(x,y)-[{\text{AO}}_{i}^{2}(g)](x,y),0]$$

$BFAO_{i}(f)$ represents the extracted bright features of the original infrared image by using the two alternating operators $AO_{i}^{1}$ and $AO_{i}^{2}$. $BFAO_{i}(g)$ represents the bright features of the original visual image extracted by using the two alternating operators $AO_{i}^{1}$ and $AO_{i}^{2}$. To produce the fusion image, the bright features of the original infrared and visual images should be combined.

Morphological operators mainly operate on the gray values of images, thus the pixel-wise comparing strategy on the gray values [4,5,6,10,25,27,32,33,38] has been an effective way for combining the image features. In this paper, this strategy is adopted for fusion the bright features of the original infrared and visual images extracted by the two types of alternating operators $AO_{i}^{1}$ and $AO_{i}^{2}$ as follows:

$$BFAO_{i}(f,\;g)\;=\;\{\begin{array}{c} {}[BFAO_{i}(f)](x,y),\\ {}[BFAO_{i}(g)](x,y), \end{array}\begin{array}{c} if\begin{array}{c} {}[BFAO_{i}(f)](x,y)> \end{array}[BFAO_{i}(g)](x,y)\\ else \end{array}$$

Similarly, for infrared image *f*, dark features having small gray values may become large after the smoothing by alternating operator $AO_{i}^{1}$ following the increasing of the scale numbers. Thus, by using the first type of alternating operator $AO_{i}^{1}$, the identified dark features of the original infrared image corresponding to scale *i* could be expressed as follows:

$$DFAO_{i}^{1}(f)\;=\;\max[[{\text{AO}}_{i}^{1}(f)](x,y)-f(x,y),0]$$

Also, by using the second type of alternating operator $AO_{i}^{2}$, the identified dark features of the original infrared image corresponding to scale *i* could be expressed as follows :

$$DFAO_{i}^{2}(f)\;=\;\max[[{\text{AO}}_{i}^{2}(f)](x,y)-f(x,y),0]$$

The dark features of the original infrared image *f* extracted by the two types of the alternating operators could be calculated as the combination of $DFAO_{i}^{1}(f)$ and $DFAO_{i}^{2}(f)$ as follows:

$$DFAO_{i}(f)\;=\;[DFAO_{i}^{1}(f)\;+\;DFAO_{i}^{2}(f)]/2$$

In the same way, the dark features of the original visual image *g* extracted by the two types of the alternating operators could be calculated as follows:

$$DFAO_{i}(g)\;=\;[DFAO_{i}^{1}(g)\;+\;DFAO_{i}^{2}(g)]/2$$

where:

$$DFAO_{i}^{1}(g)\;=\;\max[[{\text{AO}}_{i}^{1}(g)](x,y)-g(x,y),0]$$

$$DFAO_{i}^{2}(g)\;=\;\max[[{\text{AO}}_{i}^{2}(g)](x,y)-g(x,y),0]$$

Thus, based on $DFAO_{i}(f)$ and $DFAO_{i}(g)$, the dark fusion features of the original infrared and visual images extracted by the two types of alternating operators $AO_{i}^{1}$ and $AO_{i}^{2}$ are as follows.

$$DFAO_{i}(f,\;g)\;=\;\{\begin{array}{c} {}[DFAO_{i}(f)](x,y),\\ {}[DFAO_{i}(g)](x,y), \end{array}\begin{array}{c} if\begin{array}{c} {}[DFAO_{i}(f)](x,y)> \end{array}[DFAO_{i}(g)](x,y)\\ else \end{array}$$

### 4.2. Fuzzy Measure Based Final Fusion Feature Calculation

The bright fusion features $BFAO_{i}(f,\;g)$ at the *i*th scale contain the fusion features corresponding to the *i*th scale. These multi-scale bright fusion features should be combined to form the final fusion features.

These extracted fusion features are the crucial information for infrared and visual image fusion. These features contain the important spatial information of the original images. Then, the bright fusion features at any scale $BFAO_{i}(f,\;g)$, which contains more spatial information should be combined into the final fusion feature image with a larger weight.

The fuzzy theory [35,36,39] has been effectively used for image analysis applications. One image *I* with size *M ×* *N* could be treated as the fuzzy set through refining the gray value of *I* as follows:

$$\text{μ}(x,y)=I(x,y)/I_{\max}$$

where μ(x,y) represents the fuzzy value of the pixel (*x*, *y*) in image *I*. *I*_max_ represents the maximum gray value of *I*. Based on the fuzzy value μ(x,y), one fuzzy measure, linear index of fuzziness (denoted by γ) [34,37,38], could be calculated as follows:

$$\gamma (I)\;=\;\frac{2}{M\times N}\sum_{x=1}^{M}\sum_{y=1}^{N}\min\{p_{xy},(1-p_{xy})\}$$

where:

$$p_{xy}=\;\sin[\frac{\text{π}}{2}\times (1-\text{μ}(x,y))]$$

This measure, γ, using the fuzzy theory based value to calculate the contained spatial information of an image. Thus, γ could be used to construct the weight value for calculating the final fusion features.

The weight value of the bright fusion features of each scale *i* could be calculated as follows:

$$wf_{i}\;=\;\gamma [BFAO_{i}(f,g)]/\sum_{i}\gamma [BFAO_{i}(f,g)]$$

where *wf_i_* represents the weight value of the bright fusion features of scale *i*.

By using the weight value *wf_i_*, the final bright fusion features could be calculated as follows:

$$FBFAO\;(f,\;g)\;=\sum_{i}wf_{i}\times BFAO_{i}(f,g)$$

*FBFAO* (*f*, *g*) represents the final bright fusion features calculated from the multi-scale bright features by using the fuzzy measure γ. The calculation of *FBFAO* (*f*, *g*) indicates that, the bright features with more spatial information are used with a larger weight to form the final bright fusion features. Therefore, *FBFAO* (*f*, *g*) would contain more spatial information, which could produce the effective fusion image with clear regions and rich details. This would produce an effective fusion result of the original infrared and visual images. Also, the final dark fusion features could be calculated as follows:

$$FDFAO\;(f,\;g)\;=\sum_{i}df_{i}\times DFAO_{i}(f,g)$$

where:

$$df_{i}\;=\;\gamma [DFAO_{i}(f,g)]/\sum_{i}\gamma [DFAO_{i}(f,g)]$$

where *df_i_* represents the weight value of the dark fusion features of scale *i*. *FDFAO* (*f*, *g*) represents the final dark fusion features calculated from the multi-scale dark features by using the fuzzy measure γ.

### 4.3. Infrared and Visual Image Fusion

*FBFAO* (*f*, *g*) and *FDFAO* (*f*, *g*) are the final bright and dark fusion features. One direct but effective way of producing fusion image based on the extracted bright and dark fusion features is the contrast adjustment strategy [4,5,6,10,25,27,32,33,38], which could be recognized as one special type of morphological contrast operators. In this paper, we also use this strategy to import the final features into the original infrared and visual images to produce the final fusion image as follows:

*F=B* × *w*_1_*+FBFAO* (*f*, *g*) × *w*_2_ – *FDFAO* (*f*, *g*) × *w*_3_

where *B* is the base image which contains the basic information of the original infrared and visual images. Usually, *B* could be calculated as the mean of the original infrared and visual images [4,5,6,10]. *F* is the final fusion image. *w*_1_, *w*_2_ and *w*_3_ are the weights which are used to adjust the contrast of the final fusion image.

In this expression, the bright image features are added on and the dark image features are subtracted from the base image, which would not only combine the image features of the original images into the final fusion image, but also further enhance the image features. Therefore, the proposed algorithm would be effective for infrared and visual image fusion.

### 4.4. Parameter Analysis

Structuring elements, scale number *n*, *w*_1_, *w*_2_ and *w*_3_ are the main parameters used in the proposed algorithm. Because the flat structuring element is simple and easy to implement, the flat structuring element is used in this paper. In flat structuring element, the size of the structuring element at each scale is valued as the size of the corresponding scale. The shape of the structuring element is the square shape which has been recognized as the simple, effective and widely used shape in mathematical morphology [4,5,6,10]. Because the image details usually exist at the low scales [4,5,6,10], there is no need to use many scales. Usually, using 3~5 scales are enough. In this paper, we use *n* = 3 scales.

*w*_1_, *w*_2_ and *w*_3_ are the positive values used to adjust the contrast of the final fusion image, which could be valued in the interval [0,5]. To obtain an effective fusion image with good contrast, *w*_2_ and *w*_3_ should be large. Also, to keep the basic information of the original infrared and visual images, *w*_1_ should be close to 1. To be simple, we use *w*_1_ = 1.0, *w*_2_ = *w*_3_ = 2.0 in this paper. Experimental results on different types of infrared and visual images verified that the proposed algorithm using these parameters was effective.

## 5. Experimental Results

### 5.1. Visual Comparisons

To show the effective performance of the proposed algorithm for infrared and visual image fusion, experiments comparing it with the multi-scale top-hat transform-based algorithm (MSTHT) [10], multi-scale shift invariant discrete wavelet transform-based algorithm (SIDWT) [15,25], multi-scale Laplacian pyramid-based algorithm (LP) [22,25], multi-scale center-surround top-hat transform-based algorithm (MSNTHT) [6] and multi-scale toggle operator-based algorithm MSTOOC [4] are performed. SIDWT and LP are multi-scale theory-based algorithms, which could perform effectively for infrared and visual image fusion. MSTHT, MSTHST, MSNTHT and MSTOOC are multi-scale morphological theory-based algorithms, which could be effectively used for infrared and visual image fusion. The proposed algorithm is the multi-scale theory-based algorithm using morphological operators and is effective for infrared and visual image fusion. Therefore, MSTHT, SIDWT, LP, MSTHST, MSNTHT and MSTOOC are appropriate algorithms for the comparison.

The data sets used are standard data sets for infrared and visual image fusion, which could be downloaded from www.imagefusion.org. The sizes of these images range from 360 × 270 to 512 × 512. The images in the data sets are obtained under different environments. For example, the UNcamp images contain the natural and building background and the people target region is a protruding region. Also, the Dune images contain a wild background and a protruding people target region. The Navi images are obtained from the sensors located on a helicopter. Using these data sets obtained from different environments would be reasonable to verify the effectiveness of the fusion algorithms.

Some examples are shown below. In these examples, (a) is the original infrared image; (b) is the original visual image; (c) is the fusion result of MSTHT; (d) is the fusion result of SIDWT; (e) is the fusion result of LP; (f) is the fusion result of MSTHST; (g) is the fusion result of MSNTHT; (h) is the fusion result of MSTOOC; (i) is the fusion result of the proposed algorithm.

**Figure 1:**
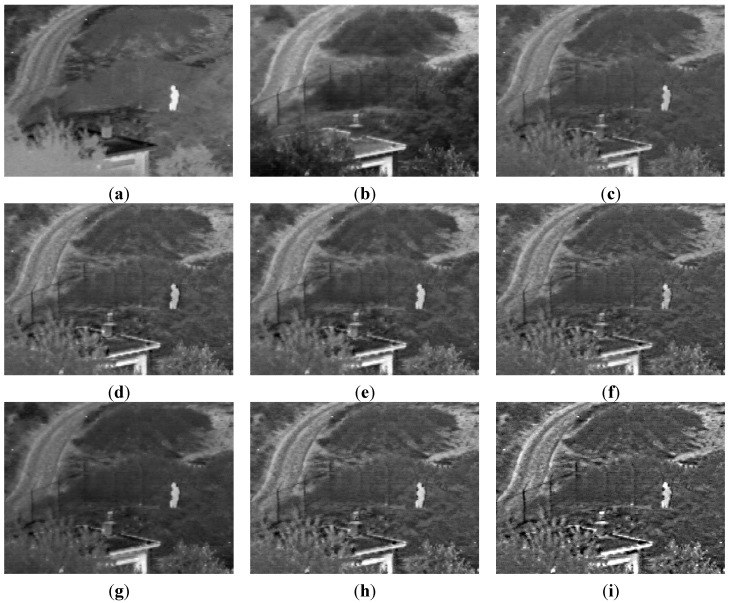
An example on UNcamp images. (**a**) Original infrared image (**b**) Original visual image (**c**) Result of MSTHT; (**d**) Result of SIDWT (**e**) Result of LP (**f**) Result of MSTHST; (**g**) Result of MSNTHT (**h**) Result of MSTOOC (**i**) Result of the proposed algorithm.

Figure 1 is an example of infrared and visual image fusion of the UNcamp images. Infrared and visual image fusion should effectively combine the image regions and details in the original images into the final fusion image. Thus, the fusion image should be clear and contain rich image details, which is useful for the further image analysis. Because some details are still smoothed, the results of MSTHT, MSTHST and MSNTHT are not clear and the details of the results of SIDWT and LP are also not clear and even worse than the result of MSTHST. The result of MSTOOC contains more details than the results of MSTHT, SIDWT, LP, MSTHST and MSNTHT, which is clearer. However, the result of the proposed algorithm is the clearest and it contains the richest image details. Therefore, the proposed algorithm performs better for infrared and visual image fusion than other algorithms.

**Figure 2:**
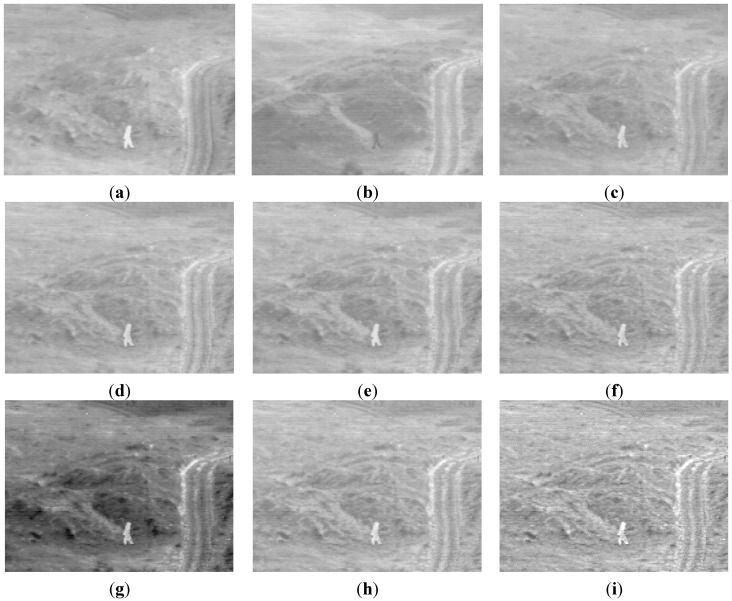
An example on Dune images. (**a**) Original infrared image (**b**) Original visual image (**c**) Result of MSTHT; (**d**) Result of SIDWT (**e**) Result of LP (**f**) Result of MSTHST; (**g**) Result of MSNTHT (**h**) Result of MSTOOC (**i**) Result of the proposed algorithm.

Figure 2 is an example of infrared and visual image fusion of the Dune images. The original images are not clear. Although MSTHT, SIDWT and LP combine the original infrared and visual images, some details are still smoothed, which results in a not very clear image. The result of MSNTHT is clearer than MSTHT, and the contrast is good, but the details are still not very clear. The results of MSTHST and MSTOOC are good and the details are clear. However, comparing with the result of the proposed algorithms, the details of the results of MSTHST and MSTOOC are not very clear. Especially, the details in the result of the proposed algorithm are very rich and the result is clearer than the results of other algorithms. These indicate the better performance of the proposed algorithm.

**Figure 3:**
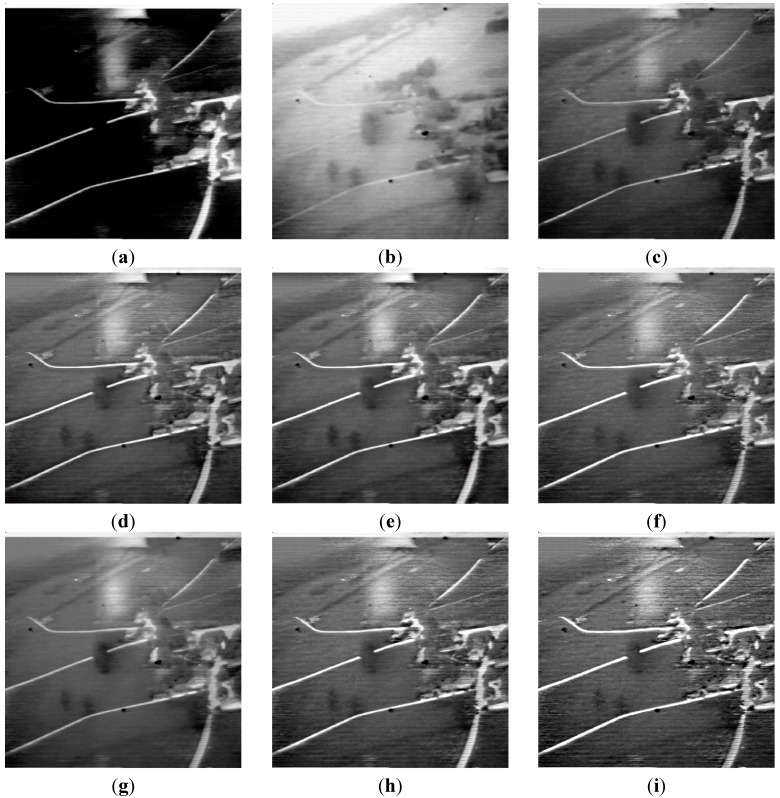
An example on Navi images. (**a**) Original infrared image (**b**) Original visual image (**c**) Result of MSTHT; (**d**) Result of SIDWT (**e**) Result of LP (**f**) Result of MSTHST; (**g**) Result of MSNTHT (**h**) Result of MSTOOC (**i**) Result of the proposed algorithm.

Figure 3 is an example of infrared and visual image fusion performed on the Navi images. The details in the original images are not clear. It would be important to produce a clear fusion result with rich details. The details of the result of MSTHT are not clear, thus the fusion result is unclear. The result of MSNTHT is better than MSTHT, but the image details are still not clear. The details of the results of SIDWT, LP and MSTHST are clearer than MSTHT and MSNTHT, but the details in the result of MSTOOC are clearer than SIDWT, LP and MSTHST. Moreover, among these algorithms, the result of the proposed algorithm is the clearest and the details are very rich, which indicates its effective performance for infrared and visual image fusion. This would be very useful for the further image analysis.

Figure 1, Figure 2 and Figure 3 indicate that the results of the proposed method contain the richest details. The fusion result of the proposed algorithm contains both the spatial information of the original infrared and visual images. The proposed algorithm not only combines these features, but also enhances these features. The high-pass filter may enhance the features, but may not perform the fusion.

Although MSNTHT combines the features of the original images, the details are not very rich. Moreover, the result of MSNTHT is closer to one of the original images (Figure 1 and Figure 3) or the contrast of the result of MSNTH is very different from the original images (Figure 2), which means MSNTHT may not preserve well the information of the original images in the final fusion image.

Infrared and visual images obtained under different environments are used to verify the effective performance of the proposed algorithm. The results show that the proposed algorithm could effectively combine the image details and regions of the original images into the final fusion image, providing a clear fusion result with rich image details.

### 5.2. Quantitative Comparisons

To show the effective performance of the proposed algorithm through a quantitative comparison, widely used measures, including entropy [6,40,41], spatial frequency [42], mean gradient [25,43] and Q measure [25,44], are adopted in this paper.

Entropy is a widely used measure to quantify the information content of an image. The fusion result of infrared and visual images contains the information of both original images. Thus, the entropy could be used as one measure to quantify the performance of the fusion algorithms. A large value of the entropy means the corresponding fusion result contains rich information, which indicates a good performance of the corresponding algorithm for infrared and visual image fusion.

Spatial frequency is defined based on the contained spatial information in an image. Fusion of infrared and visual images would combine the image regions and details of the original images into the final fused image. The fused image has clear details and should contain more spatial information. Therefore, using spatial frequency as a quantitative measure is appropriate. A large value of the spatial frequency indicates a good performance of the corresponding algorithm for infrared and visual image fusion.

Mean gradient is calculated based on the spatial gradient information. Infrared and visual image fusion should effectively combine the spatial information and produce a clear fusion result, so the mean gradient would be also an appropriate measure in the quantitative comparison. A large mean gradient value indicates a good performance of the corresponding algorithm for fusion.

Q measure has been widely used to quantify the quality of an image. An effective algorithm for infrared and visual image fusion should produce a fusion image with good quality. Thus, Q measure could be also an appropriate measure to do a quantitative comparison. Also, a large value of Q measure indicates a good fusion performance.

**Figure 4:**
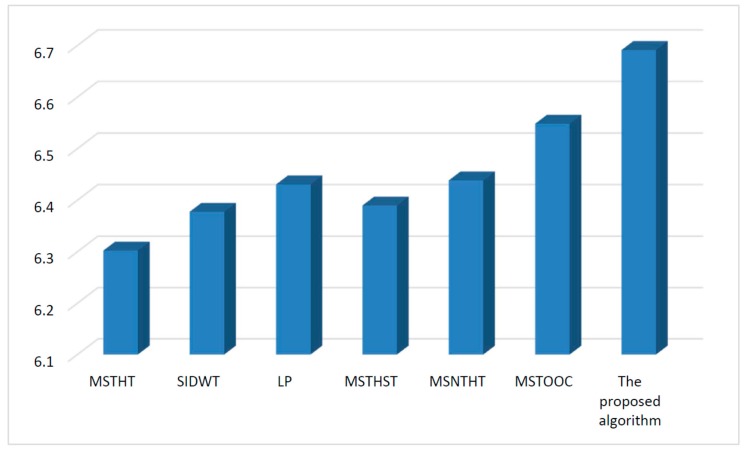
Quantitative comparison using measure entropy.

**Figure 5:**
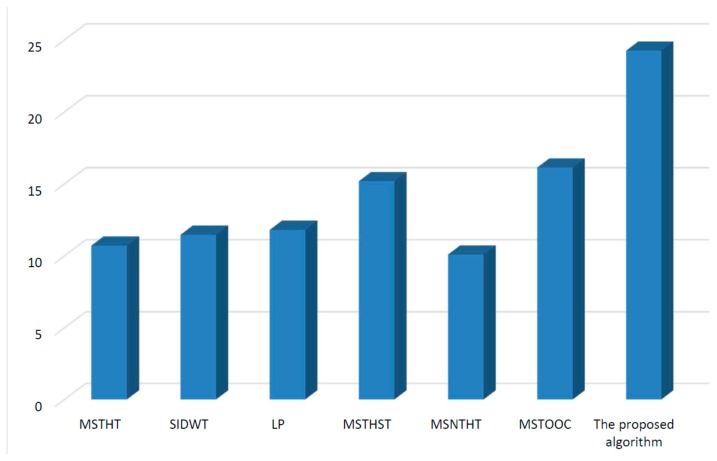
Quantitative comparison using measure spatial frequency.

**Figure 6:**
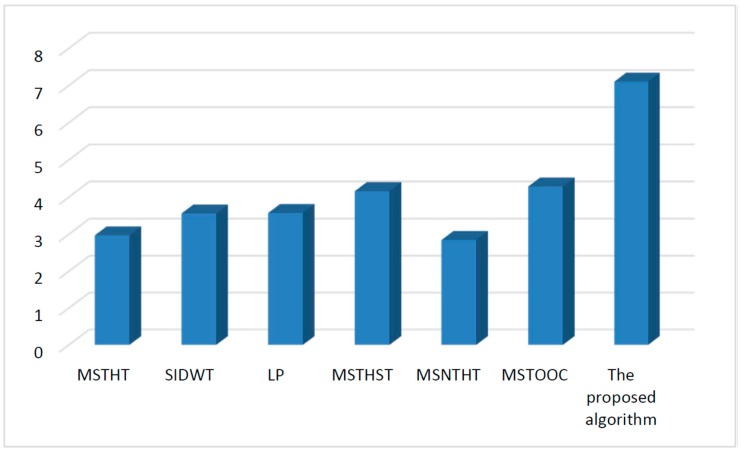
Quantitative comparison using measure mean gradient.

**Figure 7:**
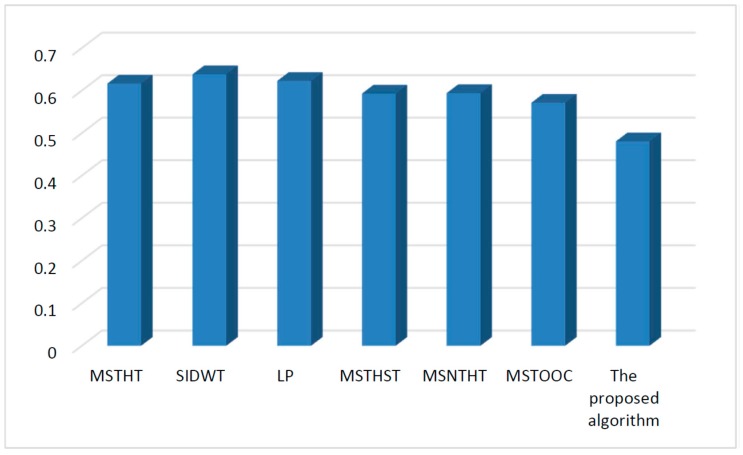
Quantitative comparison using measure Q.

Infrared and visual images obtained under different environments are processed by different algorithms. The mean value of the entropy, spatial frequency, mean gradient and Q measure values of all the fusion results related to each algorithm is shown in Figure 4, Figure 5, Figure 6 and Figure 7, respectively. 

Figure 4 shows that, the entropy value of the proposed algorithm is larger than that of the other algorithms. This means the fusion result of the proposed algorithm contains more information than those of the other algorithms, which would provide a more effective fusion result for further image analysis. Thus, the proposed algorithm performs better than other algorithms. 

Figure 5 shows that the spatial frequency value of the proposed algorithm is the largest, which verifies that the proposed algorithm based on the constructed alternating operator could give clear fusion results with rich image details.

Also, in Figure 6, the value of mean gradient of the proposed algorithm is the largest. This means the proposed algorithm combines the region and details of original infrared and visual images, which produces effective and clear fusion results.

In Figure 7, although the value of the Q measure of the proposed algorithm is not large compared with other algorithms, the value is not very different from the values of the other algorithms. This indicates the quality of the fusion images of the proposed algorithm is also good. More importantly, the values of the proposed algorithm in Figure 4, Figure 5 and Figure 6 are very larger than the values of other algorithms. Thus, in all, the performance of the proposed algorithm for fusion is effective for infrared and visual images. Therefore, because of the effective feature extraction by the constructed alternating operator and the fusion of the multi-scale features using the fuzzy measure, the proposed algorithm performs effectively for infrared and visual image fusion.

To quantitatively compare the processing times, all the algorithms are performed on images of 360 × 270 size using a computer equipped with an Intel Pentium 4, 2.6 GHz CPU and 512 MB of memory. The mean processing time of each algorithm is listed in Table 1. In Table 1, the processing times of MSTHT, SIDWT and LP are shorter than other algorithms, because the calculation of the pyramid- based multi-scale theory in SIDWT and LP is faster. Also, because the morphological operator in MSTHT is simple, the processing time of MSTHT is shorter than MSTHST, MSNTH, MSTOOC and the proposed algorithm. Especially, as the calculation of the center-surround top-hat transform used in MSNTH is time-consuming, the processing time of MSNTH is the longest. Because the alternating operators in the proposed algorithm are complicated, the processing time of the proposed algorithm is longer than MSTHT, SIDWT and LP, but the processing time of the proposed algorithm is shorter than MSTHST, MSNTH and MSTOOC. More importantly, the visual and quantitative comparisons verified that the performance of the proposed algorithm for infrared and visual image fusion was more effective than that of the other algorithms, therefore, the proposed algorithm performs effectively overall.

**Table 1:** Processing time comparison (s).

MSTHT	SIDWT	LP	MSTHST	MSNTH	MSTOOC	Proposed Algorithm
0.923	0.733	0.082	3.874	25.450	8.814	**1.828**

## 6. Conclusions

Extracting the features of the original infrared and visual images to form a clear fusion image is a crucial task. This paper proposes an effective algorithm for infrared and visual image fusion based on the fuzzy measure and the alternating operators constructed by opening and closing-based toggle operators. The extraction of the multi-scale features of the original infrared and visual images for fusion by using two types of the constructed alternating operators is discussed in detail. Also, the extracted multi-scale features are combined through the fuzzy measure-based weight strategy to form the final fusion features. In the end, the effective fusion result is produced through importing the final fusion features into the original infrared and visual images using the contrast enlargement strategy.

Because the toggle operator using opening and closing as primitives could identify the important features in the original infrared and visual images, the alternating operators could extract the features for infrared and visual image fusion well, and two types of alternating operators are used for feature extraction, which could strengthen the performance of the proposed algorithm for infrared and visual image fusion. Moreover, the strategy of combining the multi-scale features through the fuzzy measure could produce the final fusion features with rich spatial information, which would be useful for preserving the details and important regions of the original infrared and visual images in the final fused image. All of these features and the experimental results indicate that, the proposed algorithm is effective for infrared and visual image fusion, which may be also used well for other image analysis applications.
